# Interobserver, intraobserver, and interlaboratory variability in reporting pT4a colon cancer

**DOI:** 10.1007/s00428-019-02663-0

**Published:** 2019-10-16

**Authors:** Charlotte E. L. Klaver, Nicole Bulkmans, Paul Drillenburg, Heike I. Grabsch, Nicole C. T. van Grieken, Arend Karrenbeld, Lianne Koens, Ineke van Lijnschoten, Jos Meijer, Iris D. Nagtegaal, Xavier Sagaert, Kees Seldenrijk, M. F. van Velthuysen, Annette H. Bruggink, Pieter J. Tanis, Petur Snaebjornsson

**Affiliations:** 1 Department of Surgery, Amsterdam UMC, University of Amsterdam, Meibergdreef 9, 1105 AZ Amsterdam, The Netherlands; 2 Department of Pathology, Spaarne Gasthuis, Hoofddorp, Netherlands; 3 Department of Pathology, OLVG, Amsterdam, Netherlands; 4 Department of Pathology, GROW School for Oncology and Developmental Pathology, Maastricht UMC+, and Pathology & Tumor Biology, Leeds Institute of Cancer and Pathology, University of Leeds, Leeds, UK; 5 Department of Pathology, Amsterdam UMC, VU University, Amsterdam, Netherlands; 6 Department of Pathology, UMCG, Groningen, Netherlands; 7 Department of Pathology, Amsterdam UMC, University of Amsterdam, Amsterdam, Netherlands; 8 PAMM, Eindhoven, Netherlands; 9 Pathology DNA location Rijnstate, Arnhem, Netherlands; 10 Department of Pathology, Radboud UMC, Nijmegen, Netherlands; 11 Department of Pathology, UZ Leuven, Leuven, Belgium; 12 Pathology DNA, Location St. Antonius Hospital, Nieuwegein, Netherlands; 13 Department of Pathology, Erasmus MC, Rotterdam, Netherlands; 14 PALGA, the nationwide network and registry of histo- and cytopathology in the Netherlands, Utrecht, Netherlands; 15 Department of Pathology, The Netherlands Cancer Institute, Amsterdam, Netherlands

**Keywords:** Interobserver variability, T4 colon cancer, peritoneal tumor involvement

## Abstract

Clinical significance of the pT4 category in colon cancer is increasing with several therapeutic implications. The aim of this study was to evaluate variability in diagnosing pT4a colon cancer. Twelve pathologists classified 66 preselected scanned Hematoxylin/Eosin-stained slides with tumor cells at a distance of 25–1500 μm (*n* = 22), 0–25 μm (*n* = 22), or on (*n* = 22) the peritoneal surface. *Inter*- and *intra*observer variability were calculated using Kappa statistics. For *inter*laboratory variability, pathology reports of pT3 and pT4a colon cancer were extracted from the Dutch Pathology Registry between 2012 and 2015. The proportion of pT4a (pT4a/(pT3+pT4a)) was compared between 33 laboratories. Potential risk of understaging was assessed by determining the average number of blocks taken from pT3 and pT4a N0-2M0 tumors with metachronous peritoneal metastasis. *Inter*observer variability among 12 pathologists was 0.50 (95%CI 0.41–0.60; moderate agreement). *Intra*observer variability (8 pathologists) was 0.71 (substantial agreement). A total of 7745 reports with pT3 or pT4aN0-2M0 colon cancer from 33 laboratories were included for *inter*laboratory analysis. Median percentage of pT4a was 15.5% (range 3.2–24.6%). After adjustment for case mix, 8 labs diagnosed pT4a significantly less or more frequently than the median lab. Metachronous peritoneal metastases were histologically verified in 170 of 6629 pT3 and in 129 of 1116 pT4a tumors, with a mean number of blocks of 4.03(SD 1.51) and 4.78 (SD 1.76) taken from the primary tumors, respectively (*p* < 0.001). A substantial variability in diagnosing pT4a colon cancer exists, both at pathologist and laboratory level. Diagnosis of pT4a stage appears to be challenging and there is a need for standardizing assessment of this pathological entity.

**Electronic supplementary material:**

The online version of this article (10.1007/s00428-019-02663-0) contains supplementary material, which is available to authorized users.

## Introduction

A considerable group of colon cancer patients presents with T4 tumors. The T4 category represents the most advanced category with respect to local invasion [1] and is related to a high risk of developing peritoneal metastases [2, 3]. Pathological (p)T4 includes two main entities of locally advanced growth, categorized as pT4a (peritoneal penetration) and pT4b (adjacent organ/structure invasion) according to the TNM 8th ed. [4]. Intensified treatment strategies for patients with pT4 colon cancer are currently under investigation, including adjuvant hyperthermic intraperitoneal chemotherapy (HIPEC) and second-look laparoscopy, aiming at prevention and early detection of peritoneal metastases [5–7]. Furthermore, current clinical guidelines recommend adjuvant chemotherapy in stage II colon cancer in the presence of pT4 [8]. Based on a recent pooled analysis from six clinical trials, pT4 is now used to inform the duration of adjuvant chemotherapy in stage III colon cancer [9]. Thus, the pT4 diagnosis is becoming an increasingly important parameter for patient management.

Parameters used for clinical decision-making should be reliable and reproducible. Pathologists have been aware of the problems with regard to the pT4 category [10–12], with peritoneal penetration (pT4a) being a less straightforward diagnostic feature than often assumed. The TNM definition implies that full penetration of tumor cells through the peritoneum is required for pT4a. However, some national guidelines on histopathology reporting of colorectal cancer also allow tumors that are *close to* or *at* the peritoneal surface to be regarded as pT4a [13, 14]. This practice originates from studies showing that tumors that are *close to* or *at* the peritoneal surface, especially in combination with certain reactive changes, also carry negative prognostic impact and risk of peritoneal metastases similar to tumors showing full peritoneal penetration [11, 15]. From these data, it has been concluded that certain features could be allowed to represent pT4a in order to prevent underdiagnosis of pT4a [4, 10, 11, 15, 16]. Another difficulty with regard to the pT4a category is that microscopic detection of pT4 is dependent on macroscopic specimen evaluation and meticulous sampling of resection specimens [10, 12]. With the increasing clinical importance of the pT4a category, it should be clear how pathologists currently diagnose and define pT4a. Studies exploring current pathological practice and variability in diagnosing pT4a colon cancer are lacking.

The aim of this study was to evaluate inter- and intraobserver variability in diagnosing pT4 among pathologists on preselected slides, focusing on the distinction between deeply invasive pT3 and pT4a. Furthermore, interlaboratory variability and the average number of blocks taken from pT3 tumors compared with those from pT4a tumors for microscopic analysis in case of histologically verified metachronous peritoneal metastases were determined.

## Methods

### Inter- and intraobserver variability

A total of 66 Hematoxylin/Eosin-stained slides from colon carcinomas (one slide per case) were selected. These slides were selected by an experienced gastrointestinal pathologist (PS) based on the following criteria: category 1 (*n* = 22), tumors where the cancer cells approached the peritoneal surface with a distance of 25–1500 μm to the surface; category 2 (*n* = 22), where the cancer cells were very close to or at the peritoneal surface with a distance of 0–25 μm to the surface; category 3 (*n* = 22), tumors showing full peritoneal penetration with tumor cells being present on the surface. The slides were scanned (Leica Aperio AT2, × 20 or × 40) and displayed digitally to the pathologists using tEPIS, pathology image management and sharing (TraiT tEPIS). Twelve experienced Dutch and Belgian pathologists agreed to assess the slides. All pathologists had a special interest in gastrointestinal pathology and worked at university (*n* = 6) or non-university (*n* = 6) hospitals. The pathologists were asked to stage the cases as either pT3 or pT4a. The pathologists were blinded for any clinicopathological information and did not receive any diagnostic guidelines or upfront training on how to diagnose pT4a cancers. Pathologists were asked to specify the features they used to make their decision and to note any diagnostic problems. For the intraobserver analysis, the slides were presented to the pathologists in a different order for a second round of scoring. To reduce recall bias, a minimum time interval of 3 months between the first and second evaluation was used.

For descriptive purposes, Shepherd’s classification of local peritoneal involvement (LPI) was used to describe the cases, i.e., tumor *well off* (LPI1), *close to* (LPI2), *at* (LPI3), or *on* (LPI4) the peritoneal surface [15, 17], with or without mesothelial inflammatory, hyperplastic reactions and/or serosal ulceration (suppl. table 1).

### Interlaboratory variability and tumor sampling analysis

For the interlaboratory variability analysis and tumor sampling analysis, data were retrieved from the automated pathology archive PALGA, the nationwide network and registry of histopathology and cytopathology in the Netherlands. The PALGA database contains excerpts of pathology reports from all Dutch pathology laboratories (nationwide coverage since 1991) [18]. The scientific and privacy committee of PALGA approved the study protocol.

All pathology reports of pT3 and pT4 colon cancers reported between 2012 and 2015 were extracted from PALGA. Patient identifiable data were pseudonymized. Based on the deepest invasion described in the original reports, all tumors were reclassified as pT3 (colon cancers invading through the muscularis propria into pericolonic fat), pT4a (penetrating the serosa), and pT4b (invading adherent tissues or organs) according to TNM8 [4]. All pT4b tumor, rectal cancer, neuroendocrine tumor (NET), and other non-carcinoma and pathology reports of revised cases were excluded. Patients with distant metastases at the time of diagnosis were excluded using linkage with the Netherlands Cancer Registry [19], because T category has less therapeutic consequences in stage IV. Finally, only pT3 and pT4a tumors (pN0-2, cM0) were included for analysis. In the case of multiple synchronous primary pT3 or pT4a colon cancers, only the most advanced tumor per patient was included. Metachronous primary pT3 or pT4a colon cancers (resected in separate surgical procedures) were regarded as separate entities and included for analysis.

To explore interlaboratory variability in diagnosing pT4a, the proportion of pT4a (pT4a/(pT3+pT4a)) was determined and compared between the laboratories [20, 21]. To adjust for case mix and to detect variables that might explain variation in diagnosing pT4a between laboratories, multivariate regression analysis was performed. Proportions of pT4a were only used to display variability between the laboratories and no assumption on quality of the included laboratories was made based on pT4a proportions. According to national Dutch guidelines, at least 10 lymph nodes should be examined for adequate staging (www.oncoline.nl). Percentages of cases with ≥ 10 lymph nodes per laboratory and the number of (pT3+pT4) colectomy specimens per laboratory were compared to the proportions of pT4a cases.

To evaluate a potential risk of understaging, we hypothesized that histologically proven metachronous peritoneal dissemination of a tumor that was initially staged as pT3 could be related to a sampling error at the time of specimen dissection by which an area of peritoneal penetration might have been missed. For this analysis, patients with pT3 and pT4a N0-2M0 colon cancer who developed histologically verified metachronous peritoneal metastases were identified from the PALGA database. Peritoneal metastases were defined as peritoneal, omental, and/or ovarian metastases. The number of tissue blocks submitted per primary tumor resection specimen was retrieved from the pathology reports. In the Netherlands, grossing of colorectal specimens is performed variously by pathologists and laboratory technicians, mainly according to local protocols and also according to the Dutch colorectal cancer guideline (www.oncoline.nl, 2014). There is no national guideline on the minimum number of blocks from primary colon carcinoma.

### Statistics

Kappa statistics were performed to assess the degree of interobserver variability (two-way random single-measures intraclass correlation, ICC) and intraobserver variability (Cohen’s kappa). Based on an estimated expected kappa of 0.8, 95%CI [0.7–0.9], two-sided testing, alpha 0.05, power 0.80, 12 pathologists and a pT4a ratio of 50%, a sample size of 66 was necessary for measuring the ICC. A kappa of 0 means that the correlation is only due to chance, whereas a kappa of 1 refers to a perfect correlation. Values in between 0 and 1 can be interpreted (arbitrarily) as follows [22]: < 0, poor; 0–0.20, slight; 0.21–0.4, fair; 0.41–0.60, moderate; 0.61–0.8, substantial; and from 0.81, almost perfect.

For the interlaboratory variability analysis, laboratories with less than 50 synoptic reports of pT3 or pT4a colon cancer in the 4 years’ study period were excluded. The laboratory with the median proportion of pT4a diagnoses (pT4a/(pT3+pT4a)) served as a statistical reference point in order to explore variability (without implying the highest quality of this laboratory). Crude odds ratios (ORs) and 95% confidence intervals (CIs) were calculated using univariable logistic regression analyses. Variables were considered to be statistically significant if the 95% CI did not include 1. After checking for multicollinearity, statistically significant variables (age, sex, year of pathology report, tumor location, histological type, grade of differentiation, presence of lymphatic or vascular invasion, and lymph node (pN) status) were included in multivariable logistic regression analyses. Adjusted ORs and 95% CIs were calculated and compared between laboratories. Spearman’s rank correlation coefficient was used to correlate non-normally distributed continuous variables.

For the tumor sampling analysis, the mean number of tissue blocks of pT3 versus pT4a colon cancers was compared using Student’s *t* test for independent samples. A *p* value of < 0.05 was considered statistically significant. For normally distributed continuous variables, mean and standard deviation (SD) were reported; for non-normally distributed continuous variables, median and interquartile range (IQR) were provided. Statistical analyses were performed with SPSS version 24.

## Results

### Inter- and intraobserver variability

Between October and December 2016, 12 pathologists evaluated 66 slides (1 slide per case). The overall ICC was 0.50 (95%CI 0.41–0.60; moderate). The ICCs for pathologists working in university and non-university hospitals were 0.52 (95%CI 0.42–0.63) and 0.48, (95%CI 0.37–0.60), respectively. In 43 (65%) of the cases, a consensus was reached (arbitrarily defined as ≥ 80% agreement). Twenty-five (58%) were classified as pT3 and 18 cases (42%) as pT4a (suppl. table 2). No consensus was reached for the remaining 23 cases (35%), including 3 cases of category 1 (5%), 13 cases of category 2 (60%), and 7 cases of category 3 (32%).

Eight out of 12 pathologists re-evaluated the slides after 3 months. Cohen’s kappa for intraobserver variability for the eight pathologists was 0.43, 0.60, 0.66, 0.67, 0.75, 0.78, 0.85, and 0.93, respectively (median, 0.71), translating into a change in diagnosis in 3–30% of cases.

**Fig. 1:**
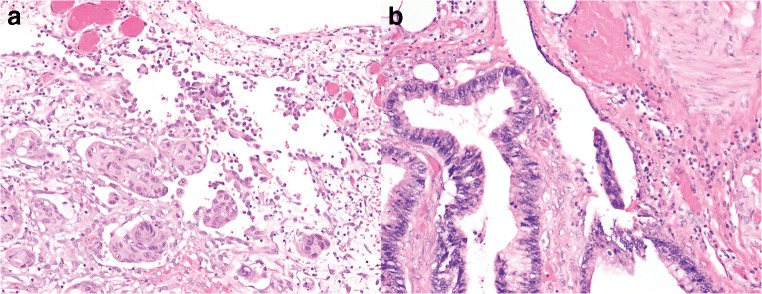
**a**, **b** Colon carcinomas with tumor cells “on” the peritoneal surface (H&E stains, × 20) that could be regarded as LPI4 (category 3). In both cases, consensus (> 80%) of pT4a was reached. However, in each case, 1 or 2 pathologists preferred pT3

**Fig. 2:**
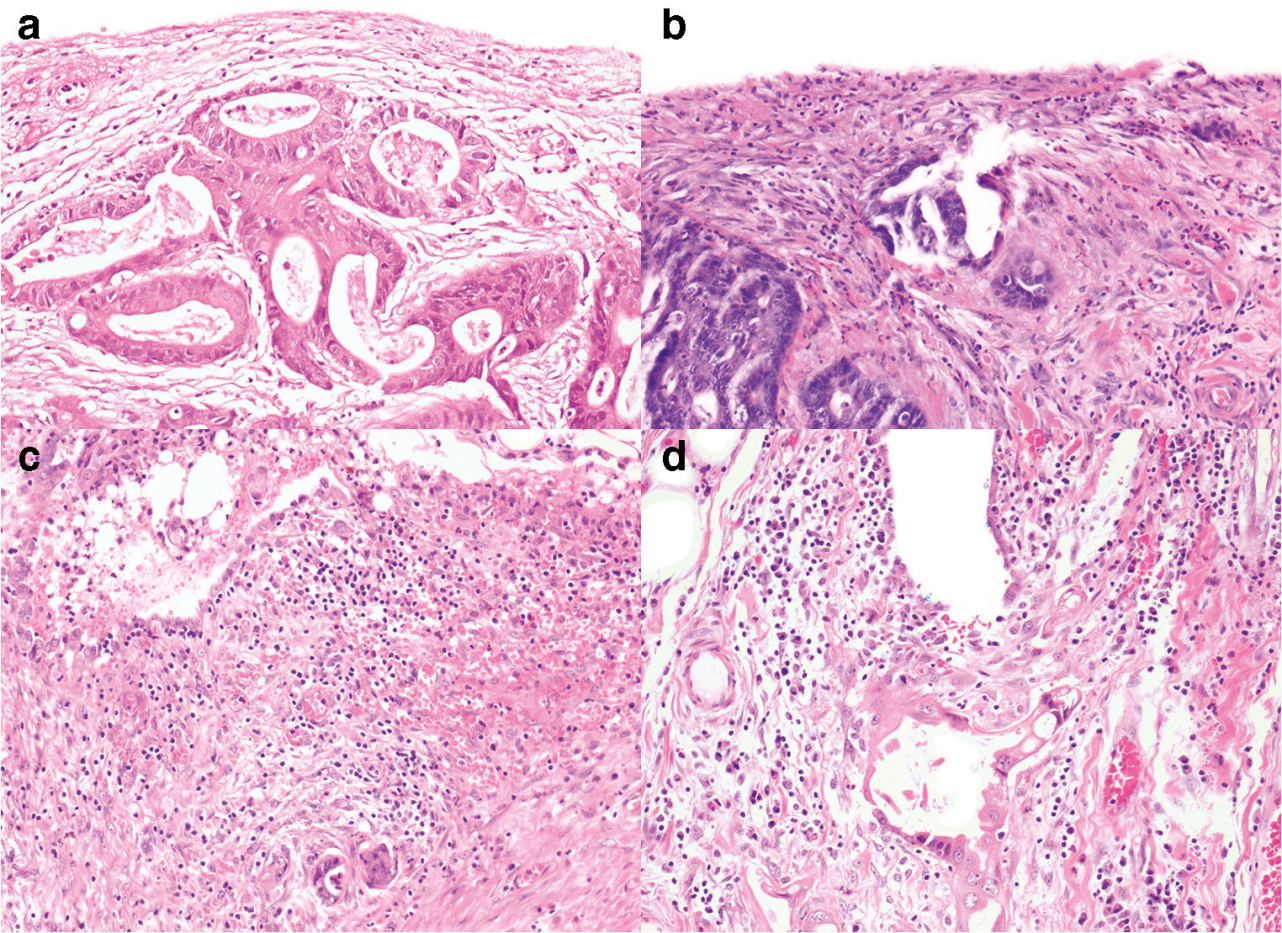
Colon carcinomas with tumor cells close to the peritoneal surface (distance to peritoneal surface, 60–200 μm (category 1); H&E stains, × 20) that could be regarded LPI2. In all cases, consensus (> 80%) of pT3 was reached; however, in all cases, still 1 or 2 pathologists preferred pT4a

**Fig. 3:**
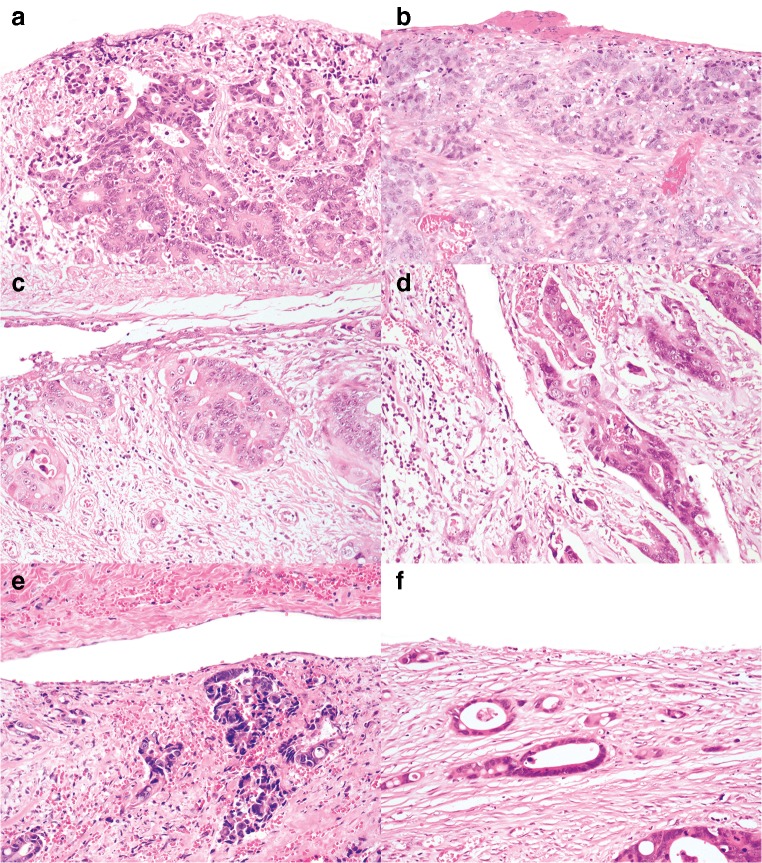
**a**–**d** Colon carcinomas with tumor cells very close to or “at” the peritoneal surface (H&E stains, × 20) that could be regarded LPI3 (distance of tumor cells to the peritoneal surface measured 25 μm or less (category 2)). No consensus was reached in cases a, b, c, and d (classified as pT4a by 4/12, 5/12, 5/12, and 9/12 pathologists, respectively). Although similar to the other cases in Fig. 3, consensus of pT3 was reached for cases e and f (considered as pT4a by 2/12 and 1/12 pathologists, respectively)

**Fig. 4:**
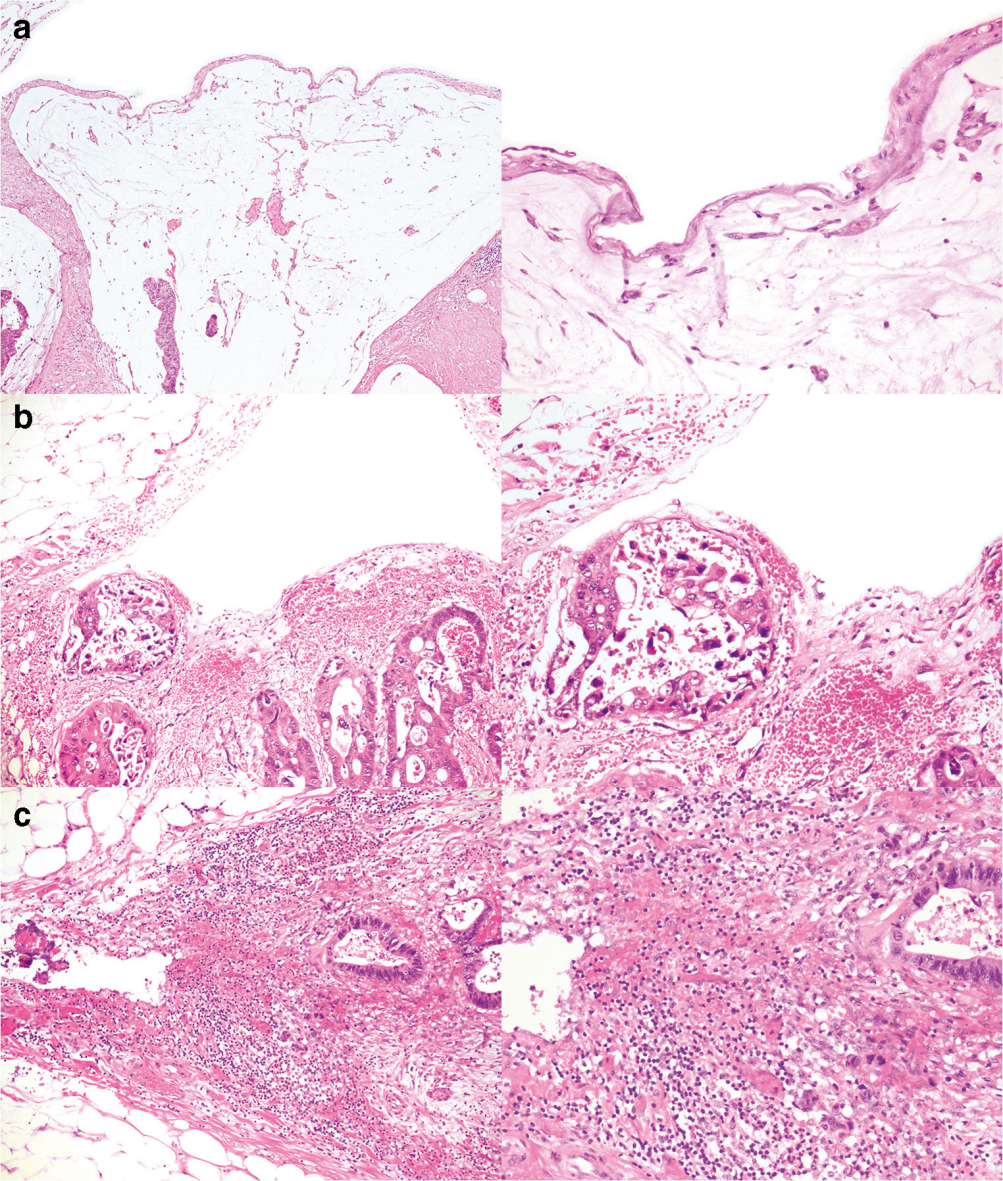
**a**–**f** Examples of cases with tumor cells or mucin close to or at the peritoneal surface with and without reactive changes (H&E stains, × 10 and × 20). In case a (classified pT4a by 2/12 pathologists), the lack of reactive changes was mentioned by some as the reason for preferring pT3. No consensus was reached in case b. Some pathologists mentioned the presence of serosal reaction, while others contradictorily described the lack of serosal reaction in this case, indicating that serosal reaction is a subjective parameter. In case c, consensus of pT3 was reached. Still, one pathologist chose T4a based on the reactive changes with the lack of a clear mesothelial lining. For other pathologists, the amount of tissue between the tumor cells and the surface was used as an argument for pT3. These cases demonstrate inconsistency in applying reactive changes when distinguishing between pT3 and pT4a

**Fig. 5:**
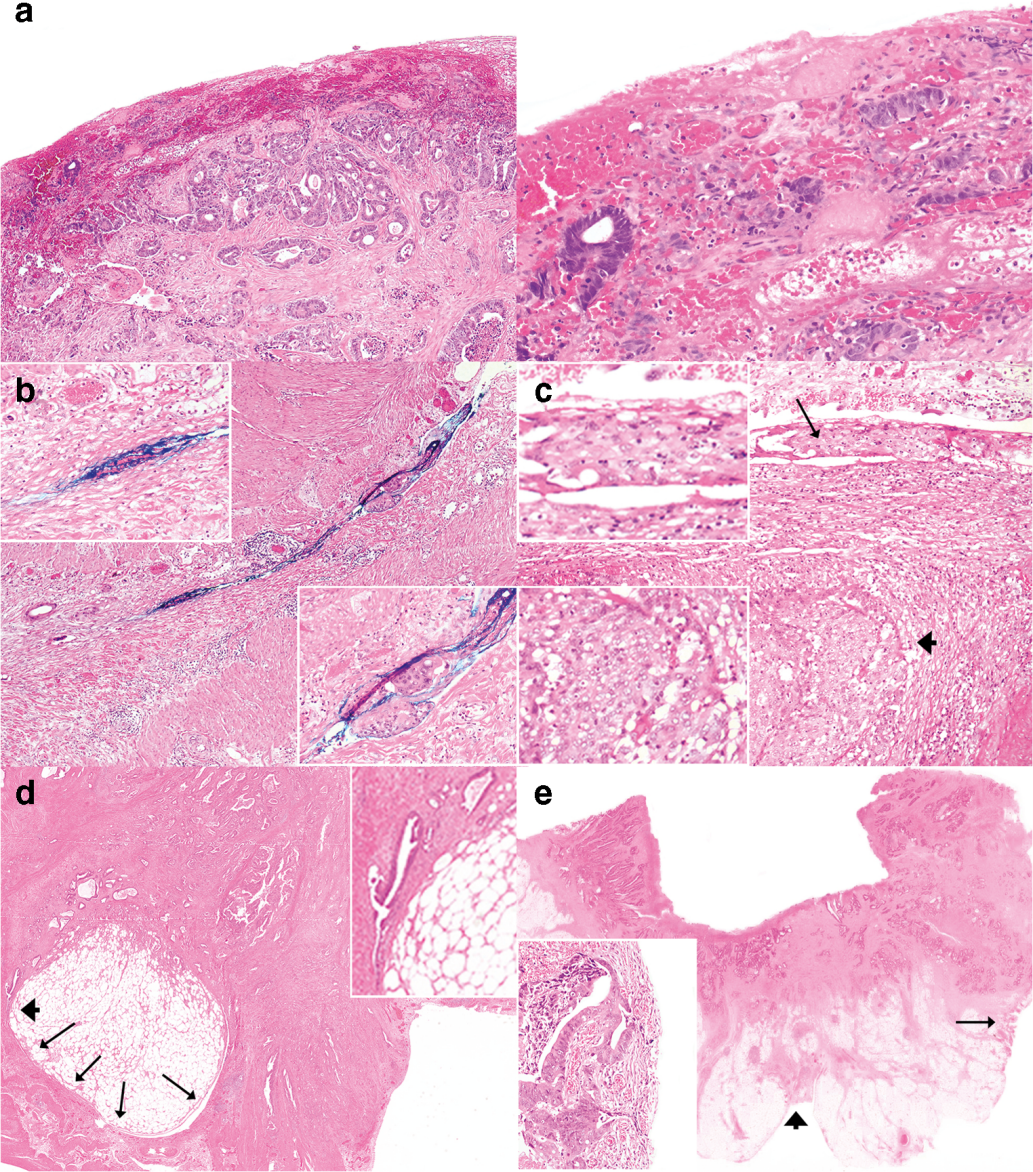
Colon carcinomas with tumor cells close to the peritoneal surface (H&E stains). Case **a**, presence of subsurface hemorrhage (× 4 and × 20) (classified pT4a by 10/12 pathologists). Case **b**, groups of tumor cells in a cleft (classified pT4a by 5/12 pathologists). By some, this was considered as a (potential) artifact. Also, case b shows ink on the surface which was mentioned as obscuring some of the morphological details, thus hindering assessment. Case **c** (× 10), reactive mesothelial cells in a cleft (arrow) resembling sheets of tumor cells that are in the vicinity (arrowhead) (classified pT4a by 3/12 pathologists). Cases **d** and **e** demonstrate peritoneal involvement that is likely to have been missed. Case **d,** peritoneal cleft buried inside the slide (arrows), focally (arrowhead) showing full penetration of tumor cells (inlet photo) (classified pT4a by 3/12 pathologists). Case **e**, most of the pathologists assessed only the peritoneal surface in front of the tumor (arrowhead) and missed the flat peritoneal surface on the side of the slide (arrow) showing tumor cells very close to the surface (classified pT4a by 2/12 pathologists)

Eight pathologists provided comments. A subsequent review of the cases and the comments highlighted several issues in differentiating between pT3 and pT4a. These issues can be broadly subdivided into the following categories: definition of the reference layer, relation of tumor cells to reference layer, reactive changes, tissue defects and artifacts, distinction between reactive mesothelial cells and tumor cells, and areas in which peritoneal penetration is easily missed. These issues are summarized in Table 1 with examples displayed in Figs. 1, 2, 3, 4, and 5.

**Table 1:** Issues in differentiating between pT3 and pT4a identified during reassessment of the slides together with the pathologists’ comments

Category	Description
Anatomical reference layer (suppl. figure 1)	Some pathologists regarded simply the surface as a reference point while others required recognizable mesothelium to be present and used that as a reference point. Others regarded the mesothelium plus a thin layer of underlying submesothelial tissue, i.e., serosal membrane, to be the reference point.
Relation of tumor cells to reference layer	Some pathologists required tumor cells to be clearly present *on* the surface for diagnosing pT4a (Fig. 1). Others considered tumor cells with variable closeness to the surface to be sufficient for pT4a (Figs. 2 and 3). Some pathologists mentioned that they regarded tumor cell growth *into* (i.e., not through) the serosal membrane as sufficient for pT4a.
Reactive changes	Some pathologists used the absence of reactive changes as an argument for pT3 (Fig. 4a), while the *presence* of reactive changes of the mesothelium and the submesothelial tissue was used as an additional argument for pT4a (Fig. 4b/c). Other pathologists did not use reactive changes when discriminating between pT3 and pT4a.
Tissue defects and artifacts	The distinction between pT3 and pT4a was sometimes hindered by various changes such as tissue damage (denuded mesothelium in Fig. 3a, b; crushed stroma in Fig. 3a; and extensive hemorrhage in Fig. 5a), leading to the choice for pT3 by default. Finally, loose groups of tumor cells, located in clefts, were by some pathologists considered peritoneal penetration while others considered these as potential artifacts (floaters) (Fig. 5b).
Distinction between reactive mesothelial and tumor cells	In case of difficulties with this distinction, the pathologists mentioned performing additional immunohistochemical stainings.
Specific areas with high chance of overlooking peritoneal penetration	In some cases areas of peritoneal tumor involvement were missed (Fig. 5d/e) frequently in clefts (Fig. 5d).

### Interlaboratory variability

**Fig. 6:**
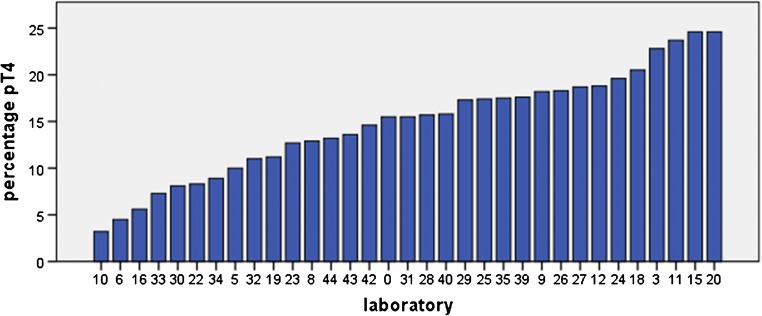
Proportion of pT4a diagnoses per laboratory for pT3-4aN0-2M0 colorectal carcinomas

After applying the predefined exclusion criteria, 7745 cases with pT3/pT4a cM0 from 33 laboratories were used for analysis (suppl. figure 2). The number of colon cancer resection specimens synoptically registered per laboratory ranged from 58 to 797 (median 209, IQR 130–278.5). The median proportion of pT4a cases was 15.5% (ranging from 3.2 to 24.6%) (Fig. 6). There was neither a linear association between the number of colectomy specimens examined and the proportion of pT4a cases (*p* = 0.310) nor between the percentage of cases with ≥ 10 lymph nodes examined and the proportion of pT4a cases (*p* = 0.282). It should be noted that a minimum threshold of 10 examined lymph nodes is used in the Netherlands (www.oncoline.nl, 2014) instead of 12 in many other guidelines.

**Fig. 7:**
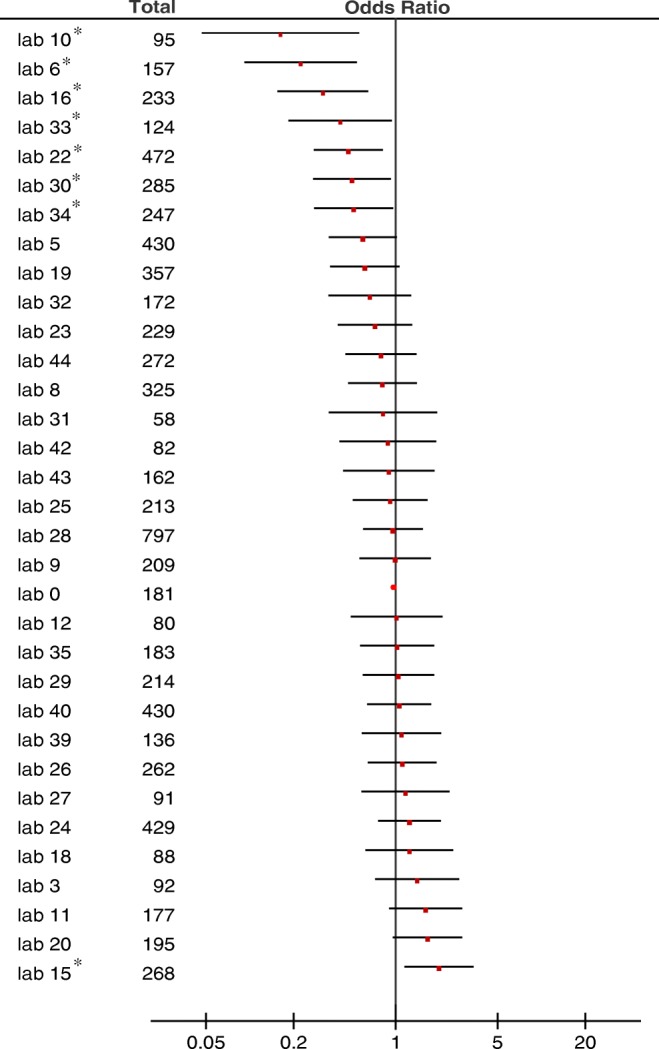
Adjusted OR’s per laboratory. Laboratory 0 is the reference laboratory based on the median. Asterisk indicates laboratories that significantly differed from the reference laboratory. Total, total numbers of pT3-4aN0-2M0 specimens evaluated in each laboratory

Factors significantly associated with a pT4a diagnosis in univariable analysis were female sex, histological type, presence of lymphatic or vascular invasion, presence of lymph node metastases, and laboratory. These factors were included in multivariable analysis to determine case mix–corrected interlaboratory variability (suppl. table 3). Adjusted ORs for the separate laboratories, compared with the reference laboratory, are shown in Fig. 7. In total, 8 laboratories (24.2%) significantly differed in diagnosing pT4a compared with the median lab after adjusting for case mix. In one laboratory, pT4a was diagnosed significantly more frequently and in seven laboratories less frequently than in the median laboratory.

### Tumor sampling analysis

Of the 7745 selected pT3/pT4a cM0 colon cancer patients, 299 (3.8%) were identified as having histologically verified metachronous peritoneal metastases at the 1st of April 2018 according to PALGA. In 170 of the 299 patients, the primary tumor was classified as pT3, in the remaining 129 patients as pT4a. Based on the PALGA database, the proportion of metachronous peritoneal metastases was 170/6629 (2.6%) for pT3 and 129/1116 (12%) for pT4a. The number of blocks taken from the primary tumor was normally distributed. In patients with metachronous peritoneal metastases, the mean number of blocks sampled in pT3 tumors was 4.03 (SD 1.51), which was significantly less than 4.78 blocks (SD 1.76) in pT4a colon cancers (*p* < 0.001). For pT3 and pT4a tumors with metachronous peritoneal metastases, the number of cases with 5 or more blocks taken from the primary tumor was 31% (53/170) and 49% (63/129), respectively (5 tissue blocks are a recommended minimum according to the UK guidelines [14]).

## Discussion

In this study, we explored the current pathological practice of diagnosing pT4a colon cancer. The main findings are a moderate *inter*observer agreement in distinguishing pT4a from deeply invasive pT3 and a substantial *intra*observer agreement. Most disagreements were found in cases with tumor cells at a distance of 0–25 μm to the surface, but even preselected slides with tumor cells on the peritoneal surface resulted in disagreement. Also, after adjustment for case mix, the proportion of pT4a colon cancers differed significantly between the median laboratory and eight other laboratories (24%). Furthermore, pT3 tumors from patients who subsequently developed peritoneal metastases were diagnosed using significantly lower number of tissue blocks than for the diagnosis of pT4a tumors from patients who developed peritoneal metastases. Our findings highlight inconsistencies in diagnosing pT4a in colon cancers. Because the pT4 category increasingly bears clinical and therapeutic consequences, there is an urgent need for a better definition of the pT4a category, which can be applied by pathologists in a reliable and reproducible fashion.

Studies evaluating the practice of diagnosing pT4a colon cancer at a pathologist and/or laboratory level are scarce. Littleford et al. [23] determined interobserver variability of the four categories of Shepherd’s LPI classification, using a single-center cohort of 138 cases of pT3/pT4 cases. Kappa values ranged between 0.45 and 0.64 among four pathologists assessing the separate LPI categories, which is similar to the present study [23]. Agreement increased when only LPI1–2 had to be discriminated from LPI3–4, with Kappa values ranging from 0.74 to 0.89.

Detailed macroscopic examination and subsequent extensive sampling of suspected areas have been described to likely improve the accuracy of pT4a assessment [12, 17], although studies on the subject are still limited. Our study is the first to show that pT4a is likely missed in some pT3 cases due to inadequate sampling. In a recent guideline from the Royal College of Pathologists, UK, it is recommended that a minimum of 5 blocks should be taken from the primary tumor for an accurate assessment of various pathology parameters [14]. Data from the present study appear to support this, with an average of approximately 5 tissue blocks being taken from primary pT4a tumors that developed peritoneal metastases, as opposed an approximate average of 4 tissue blocks from pT3 tumors that developed peritoneal metastases. Currently, the Dutch colorectal cancer guideline does not contain any recommendation on the minimum number of blocks from the primary tumor for accurate assessment of pT status. In the current study, 4 tumor blocks or less, i.e., less than the minimum of 5 blocks according to the UK guideline, were submitted in 61% of analyzed cases (183/299). Also, the low frequency of pT4a in some of the laboratories (Fig. 6) likely represents underdetection. An optimal pT4a detection rate for stage II and III tumors is not known but a threshold of 20% for pT4a+b in all stages combined has been recommended in the UK [4]. The current data raise the question if a minimum number of blocks from the primary tumor for pT assessment need to be put forward in the Dutch guidelines and implemented as an audit and quality criterion, similar to the minimum number of examined lymph nodes.

In this study, we observed different approaches among pathologists to diagnose pT3 or pT4a. Various criteria were applied, some of which being highly subjective and often inconsistently used. Most discrepancies among pathologists can be traced back to two main schools of thoughts, one of them being more strict and requiring cells to be growing through the peritoneum and be present on the peritoneal surface (Fig. 1, LPI4), while the other is less prescriptive with pT4a already being considered when tumor cells are close to or at the peritoneal surface (LPI2/3), especially in the presence of particular reactive changes.

Attempts have been made to further define the pT4a category in the literature. The main UICC and AJCC TNM definition of pT4a, i.e., *tumor that perforates visceral peritoneum*, has hardly changed during the last 20–30 years except that in the AJCC 7th and 8th edition, the word *perforates* was exchanged with the word *penetrates* and *invades*, respectively. In some national guidelines, further details on the definition of pT4a have been put forward. In both the US and UK guidelines, this is based on the LPI categorization by Shepherd et al. [15, 17]. In the College of American Pathologists guideline from 2017, the pT4a definition includes *tumor present at the serosal surface* and *free tumor cells on the serosal surface with underlying erosion/ulceration of mesothelial lining, mesothelial hyperplasia and/or inflammatory reaction* (roughly equates to LPI3 and 4) [13]. In the current UK guideline from 2018, pT4a is defined as *tumor cells visible either on the peritoneal surface, free in the peritoneal cavity or separated from the peritoneal surface by inflammatory cells only* (also roughly LPI3 and 4) [14]. In the Netherlands, the national guideline on colorectal cancer does not give any details on the definition of pT4a beyond the TNM literature (www.oncoline.nl). The LPI classification might be suboptimal because the distinction between LPI2 and LPI3 may be unclear in many cases where tumor cells approach the peritoneal surface. Also, a gradient in prognostic impact within the LPI3 category has been described [24]. Recently, some authors have proposed that colon cancers 1 mm or less from the serosal surface should be regarded as pT4a when additionally displaying certain features (serosal fibroinflammatory reaction; peritumoral abscesses that communicate with the serosa; serosal hemorrhage; and serosal fibrin) [11]. Other authors have proposed that invasion beyond the peritoneal elastic lamina should be regarded as pT4a [25]. Various proposed additional criteria for diagnosing pT4a may, however, contradict one of the general rules of the TNM system that states that *if there is doubt concerning the correct T, N or M category to which a particular case should be allotted, then the lower (*i.e.*, less advanced) category should be chosen* [4]. Variation in guidelines and literature suggestions regarding the pT4a definition has likely contributed to the confusion in what represents pT4a [26].

Defining pT4 has been mainly based on survival, and seldom on the risk of metachronous peritoneal metastases. The upcoming treatment strategies for peritoneal metastases justify consideration of the risk of peritoneal metastases when defining pT4. Shepherd [15] reported that peritoneal recurrences all occurred in the LPI3 and 4 group except for one case in the LPI2 group (1%). In a recent series [27] of 159 patients, the 5-year peritoneal recurrence rate was 33% for true peritoneal penetration (LPI4), as opposed to 21% (*p* = 0.057) for peritoneal reaction with tumor less than 1 mm from the peritoneum (LPI2–3). Using peritoneal scrape cytology [11], tumor cells were found in 46% and 55% of the deeply invasive pT3 and pT4a tumors (vs. 19% in all pT3), translating into peritoneal recurrence rates of 11% and 18%, respectively. These data show that the presence of tumor cells on the peritoneal surface carries a higher risk of peritoneal metastases than when tumor cells are close to or at the surface but without full penetration. Although the risk of local, peritoneal, or systemic recurrence is also increased in these deeply invasive pT3 cases, it remains unclear whether that justifies including them into the pT4a category.

There are some limitations related to the present study. Regarding the interobserver variability analysis, the selected samples might not have been a realistic representation of daily clinical practice, also since deeper levels and analyzing/adding more tumor sections was not possible in the present research setting, potentially leading to an underestimation of interobserver agreement. We rather choose this design in order to identify pitfalls and points of attention. In addition, virtual slide analysis requires training and may be less efficient on this kind of material. In the interlaboratory analysis, the use of the median laboratory as a reference is convenient for describing the level of variation that exists between laboratories [20, 21]. This study does not attempt to provide information on which frequency of pT4a would be optimal. Finally, the sampling analysis might be confounded by for example fewer blocks being taken from areas grossly suspicious of T4a and by variability in microscopic assessment. Despite these shortcomings, we were able to demonstrate a difference in the number of tissue block between pT3 and pT4a cases, which may even strengthen our finding. It should also be mentioned that the frequency of peritoneal metastases is most likely underreported in this series, as in daily clinical practice, not all peritoneal metastases are histologically confirmed.

We conclude that the current pathology practice leaves room for subjectivity and variable interpretation when distinguishing pT3 from pT4a colon cancer. Also, the current literature on the topic is limited and does not offer enough data on how pT3 and pT4a should be distinguished. Considering the potential therapeutic and prognostic implications, the reproducibility of pT4a diagnosis should be improved, both with regard to sampling and microscopic assessment. Especially, the gray area of peritoneal involvement should be clarified with explicit criteria to distinguish pT4a from pT3. To achieve this, future research should aim at assessing the histopathology of pT3-pT4a within clinical trials with detailed follow-up regarding peritoneal recurrences.

## Electronic supplementary material

ESM 1 (DOCX 711 kb)
