# Supplementary material for: Interobserver, intraobserver, and interlaboratory variability in reporting pT4a colon cancer
Source: Virchows Arch. 2019 Oct 16;476(2):219–30. doi: 10.1007/s00428-019-02663-0 (PMC7028812; doi:10.1007/s00428-019-02663-0)
Supplement: Supplementary file 1 — (DOCX 711 kb) [file 428_2019_2663_MOESM1_ESM.docx]

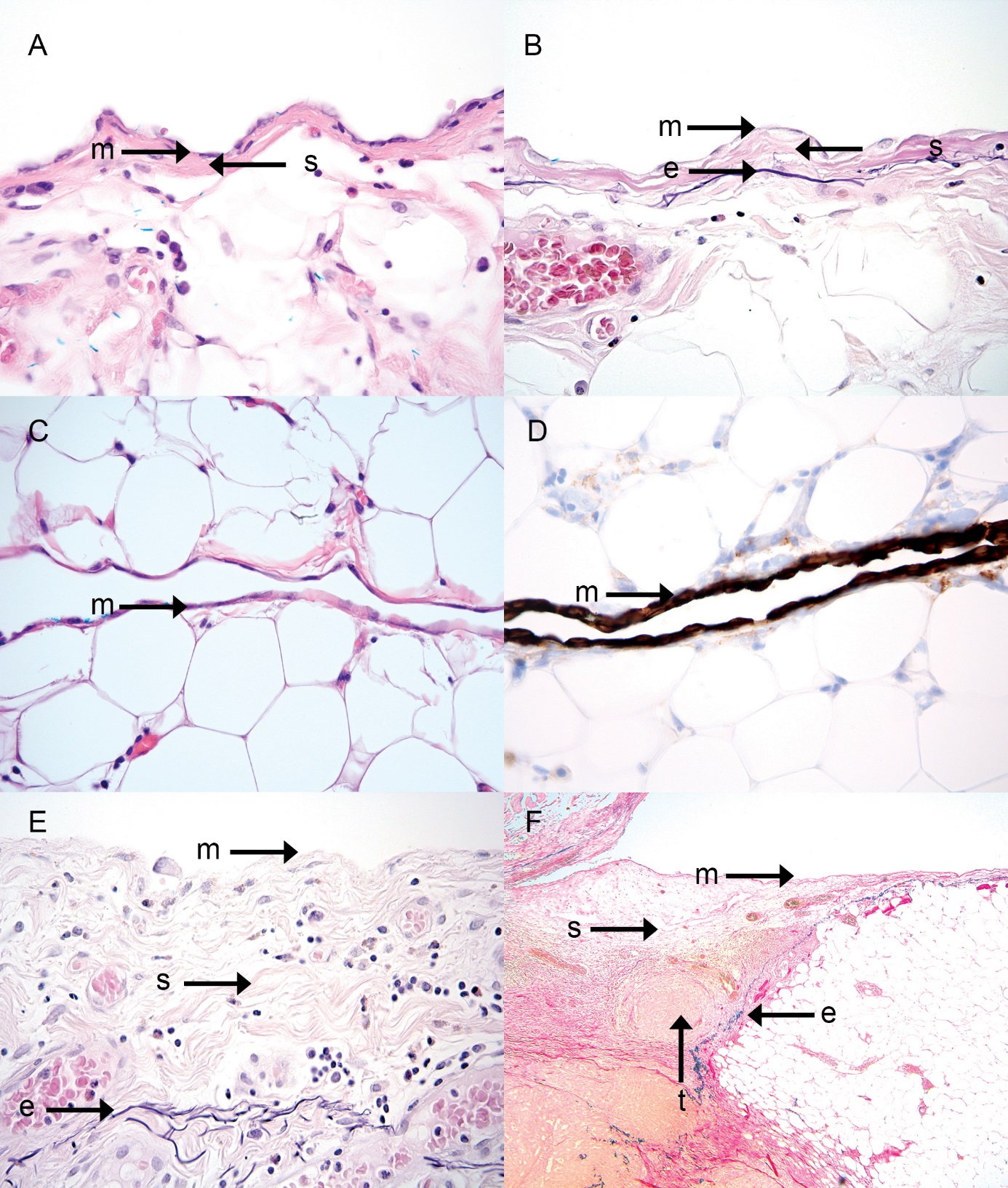
**Suppl. fig. 1** Anatomical layers of the peritoneum. Case a (H&E stain, x40) and b (Elastica van Gieson, EvG, stain, 40x) illustrate the prototypical microanatomy of the peritoneum (serosa, serous membrane), consisting of a mesothelium (m), a submesothelial stroma layer (s), and a peritoneal elastic lamina (e). The elastic fibers of the peritoneal elastic lamina stain black/dark purple in the EvG stain. Case c (H&E stain, 40x) and d (cytokeratin 7 stain, 40x) are examples of peritoneum in a cleft between fatty lobules, here consisting of a flat mesothelium with minimal submesothelial stroma and no demonstrable peritoneal elastic lamina (EvG stain not shown). The mesothelial cells are accentuated using the cytokeratin 7 stain (brown). Cases a through d demonstrate that the normal peritoneum is variously developed depending on the location. Case e (EvG stain, x40) illustrates slightly thickened submesothelial stroma layer (s) in the presence of mild inflammation. In case e the mesothelium (m) is largely stripped off. Case f (EvG stain, x10) illustrates severely thickened submesothelial stroma layer (s) due to colorectal carcinoma (t) that grows through the peritoneal elastic lamina (e) and into the thickened submesothelial stroma layer. The black stained peritoneal elastic lamina can be followed diagonally towards the right upper corner where the peritoneum is closer to normal thickness.

**Suppl. Fig. 2** Patient inclusion

| **LPI - primary tumor** | |
| --- | --- |
| LPI1 | Tumor well clear of closest peritoneal surface |
| LPI2 | Mesothelial inflammatory and/or hyperplastic reaction with tumor close to, but not at the peritoneal surface |
| LPI3 | Tumor present at peritoneal surface with inflammatory reaction, mesothelial hyperplasia and/or erosion or ‘ulceration’ |
| LPI4 | Tumor cells shown free on the serosal surface with evidence of adjacent ‘ulceration’ of the visceral peritoneum. |

**Suppl. table 1** Local Peritoneal Involvement (LPI) score according to Shepherd

| Number of pathologists with same assessment | Number of cases  n = 66 | Cases assessed as: | |
| --- | --- | --- | --- |
|  |  | pT3 | pT4a |
| 12 out of 12 pathologists (100% agreement) | 20 (30%) | 13 | 7 |
| 11 out of 12 pathologists (92% agreement) | 10 (15%) | 6 | 4 |
| 10 out of 12 pathologists (83% agreement) | 13 (20%) | 6 | 7 |
| 9 out of 12 pathologists (75% agreement) | 8 (12%) | 3 | 5 |
| 8 out of 12 pathologists (67% agreement) | 5 (8%) | 1 | 4 |
| 7 out of 12 pathologists (58% agreement) | 8 (12%) | 6 | 2 |
| 6 out of 12 pathologists (50% agreement) | 2 (3%) | NA | NA |

**Suppl. table 2** Level of agreement amongst the twelve pathologists per case

| **Suppl. table 3. Baseline characteristics and case mix correction** | | | | | | | |  |
| --- | --- | --- | --- | --- | --- | --- | --- | --- |
|  |  | | ***Total (T3+T4a)*** | | ***T4a*** | | ***OR*** | ***Adjusted OR*** |
|  |  | | *n* | % | *n* | % |  |  |
| Sex | M | | 3985 | 51.5% | 520 | 46.6% |  |  |
|  | V | | 3760 | 48.5% | 596 | 53.4% | **1.255 (1.105-1.425)** | **1.234 (1.078-1.413)** |
| Age | < 60 | | 1060 | 13.7% | 157 | 14.1% |  |  |
|  | 60-69 | | 2252 | 29.1% | 305 | 27.3% | 0.901 (0.732-1.109) |  |
|  | 70-79 | | 2640 | 34.1% | 351 | 31.5% | 0.882 (0.720-1.082) |  |
|  | 80 ≤ | | 1793 | 23.2% | 303 | 27.2% | 1.170 (0.948-1.443) |  |
| Year of examination | 2012 | | 1601 | 20.7% | 223 | 20.0% |  |  |
|  | 2013 | | 2015 | 26.0% | 271 | 24.3% | 0.960 (0.793-1.162) |  |
|  | 2014 | | 2341 | 30.2% | 354 | 31.7% | 1.101 (0.919-1.319) |  |
|  | 2015 | | 1788 | 23.1% | 268 | 24.0% | 1.090 (0.899-1.320) |  |
| Tumour location | Right hemicolon | | 4002 | 51.7% | 593 | 53.1% |  |  |
|  | Transverse colon | | 350 | 4.5% | 57 | 5.1% | 1.118 (0.831-1.505) |  |
|  | Left hemicolon | | 934 | 12.1% | 137 | 12.3% | 0.988 (0.808-1.208) |  |
|  | Sigmoid | | 2412 | 31.1% | 320 | 28.7% | 0.879 (0.760-1.018) |  |
| Histological type | Adenocarcinoma, w./m. d. | | 5834 | 75.3% | 747 | 66.9% |  |  |
|  | Adenocarcinoma, poorly d. | | 872 | 11.3% | 171 | 15.3% | **1.661 (1.382-1.997)** | **1.294 (1.059-1.580)** |
|  | Mucinous carcinoma | | 894 | 11.5% | 152 | 13.6% | **1.395 (1.153-1.688)** | **1.506 (1.231-1.843)** |
|  | Signet ring cell carcinoma | | 78 | 1.0% | 32 | 2.9% | **4.737 (2.998-7.487)** | **3.633 (2.214-5.961)** |
|  | Other carcinoma’s | | 67 | 0.9% | 14 | 1.3% | **1.799 (0.993-3.258)** | **1.819 (0.967-3.421)** |
| Lymphatic or vascular invasion | | Absent | 5539 | 71.5% | 593 | 53.1% |  |  |
|  |  | Present | 2019 | 26.1% | 484 | 43.4% | **2.630 (2.302-3.004)** | **1.839 (1.576-2.146)** |
| Lymph node status | N0 | | 4368 | 56.4% | 403 | 36.1% |  |  |
|  | N1 | | 2205 | 28.5% | 400 | 35.8% | **2.180 (1.878-2.531)** | **1.921 (1.637-2.255)** |
|  | N2 | | 1172 | 15.1% | 313 | 28.0% | **3.585 (3.040-4.228)** | **2.477 (2.047-2.997)** |

**Suppl. table 3** Baseline characteristics and case mix for pT3 and pT4a colon cancer patients. Abbreviations: adenocarcinoma w./m. d.: adenocarcinoma, well/moderately differentiated; adenocarcinoma poorly d.: adenocarcinoma poorly differentiated. OR: odds ratio.
